# Standard CMOS Fabrication of a Sensitive Fully Depleted Electrolyte-Insulator-Semiconductor Field Effect Transistor for Biosensor Applications

**DOI:** 10.3390/s90604366

**Published:** 2009-06-04

**Authors:** Gil Shalev, Ariel Cohen, Amihood Doron, Andrew Machauf, Moran Horesh, Udi Virobnik, Daniela Ullien, Ilan Levy

**Affiliations:** Intel Research Israel, Intel Electronics, Jerusalem 91031, Israel; E-Mails: gil.shalev@intel.com (G.S.); ariel.cohen@intel.com (A.C.); amihood.doron@intel.com (A.D.); andrew.machauf@intel.com (A.M.); moran.horesh@intel.com (M.H.); udi.virobnik@intel.com (U.V.);daniela.ullien@intel.com (D.U)

**Keywords:** EISFET, biosensors, CMOS, fully depleted

## Abstract

Microfabricated semiconductor devices are becoming increasingly relevant for detection of biological and chemical components. The integration of active biological materials together with sensitive transducers offers the possibility of generating highly sensitive, specific, selective and reliable biosensors. This paper presents the fabrication of a sensitive, fully depleted (FD), electrolyte-insulator-semiconductor field-effect transistor (EISFET) made with a silicon-on-insulator (SOI) wafer of a thin 10-30 nm active SOI layer. Initial results are presented for device operation in solutions and for bio-sensing. Here we report the first step towards a high volume manufacturing of a CMOS-based biosensor that will enable various types of applications including medical and environmental sensing.

## 1. Introduction

In recent years, research and development of biosensors has received a great deal of attention since their extensive application potential is highly recognized in areas such as medical diagnostics and the food industry [1-4]. Biosensors are normally composed of two main components - the sensing device and the sensing molecule, i.e. chemical or biological recognition elements. As a functional hybrid system, the biggest challenge is to optimize this system to benefit from coupling of the unique features of the bio-recognition event with the sensitive signal recognition and amplification potential of a sensing device [5].

One of the major classes of silicon-based sensors uses a field-effect transistor (FET) as the transduction element for the analytical signals. In its basic configuration, the FET sensor is a field-effect transistor where the metal gate electrode is replaced by an ion-conducting solution and a reference electrode. This Electrolyte-Insulator-Semiconductor FET is commonly referred to as an EISFET. An inorganic dielectric material is used as the interface between the device and the solution, and its electrical response is sensitive to ion concentration in the solution [6-9]. This ion-sensitive field-effect transistor (ISFET) was first introduced by Bergveld in 1970 [10]. Eventually a membrane or other element can be added to the dielectric material to couple the biological components and to induce selectivity towards specific analytes [11]. Among various types of transducers used for biosensing, EISFET, along with its many inherent drawbacks is still one of the most investigated devices for electronic biosensing [12,13]. During the past 30 years, EISFET technology and applications have achieved a remarkable level of development [14]. FET-related devices have appeared with molecular selectivity (enzyme sensors [15], immunosensors [16] and DNA sensors [17]) and even with the ability of measuring complex biological receptors and cells [18]. Therefore, achieving an optimized sensor behavior in EISFETs usually requires the use of specific materials and device architectures.

The ability to detect biomolecular interactions is of extreme importance in medical diagnostics. Nevertheless, it also often requires single-use disposable sensors that would be fabricated in high volumes and will require a very low unitary cost. Much effort is being laid on nanowire-based FETs for biosensing. However, these nanowire-based devices suffer from very low manufacturing potential [19-21]. On the other hand, silicon microelectronic technology can provide a low cost manufacturing infrastructure for a high volume fabrication, but this requires the use of standard manufacturing process. Here we describe a standard CMOS manufacturing of an EISFET. The EISFET reported here comprises a thin conducting layer of 10 – 30 nm Silicon-On-Insulator (SOI) which implies that the active silicon is fully depleted (FD) for the given silicon doping. It was already demonstrated that FD EISFETs present enhanced electrical performance in terms of increased sensitivity to surface potential variations [22,23]. This sensitive device holds the potential application in medical diagnostics for biomarker analysis.

## 2. Experimental

### 2.1. Device Fabrication

6″ Silicon-on-Insulator (SOI) wafers were used (SOITEC, Bernin, France). SOI layer and buried-oxide (BOX) thickness was 260 nm and 1,000 nm, respectively, while SOI resistivity was 13–22 Ωcm. Two types of devices were fabricated under the same process: FD EISFETs and metal-oxide-semiconductor FET (MOSFET) like devices that serve as test structures for process evaluation and electrical definition. Henceforth, these MOSFETs will be referred to as test structures. The fabrication of the devices was as follows: SOI layer was thinned down using oxidation and oxide removal. Several consecutive oxidation/oxide-removal steps took place in order to ensure a small thickness variation across the wafers. Eventually, several wafers with SOI thickness in the 10 – 30 nm range were fabricated with with-in-wafer SOI thickness variation not greater than 10%. The SOI EISFET is fully depleted (FD) for the given SOI resistivity (doping) and SOI thickness. MESA-type isolation was used between the devices. Subsequent arsenic implant (15 keV/5 e14)) for the source and drain regions took place followed 100 nm SiO_2_ PECVD for inter layer dielectric (ILD) and opening of the contacts. Ti/Al/TiN was sputtered and patterned for interconnection purposes followed by 4,500 Å passivation layer of PECVD nitride. The seed Ti/Au layer was sputtered followed by Au electroplating in the pad areas. The metal gate of the MOSFETs is located over a 100 nm PECVD SiO_2_ layer which is significantly thicker than the 30 nm LPCVD SiO_2_ of the FD EISFETs. The last step of the process was the actual opening of the passivation above the FD EISFETs' active region. This was performed with dry etch followed by final wet etch in order to ensure no physical and/or electrical damage to the underlying active gate.

**Figure 1.**
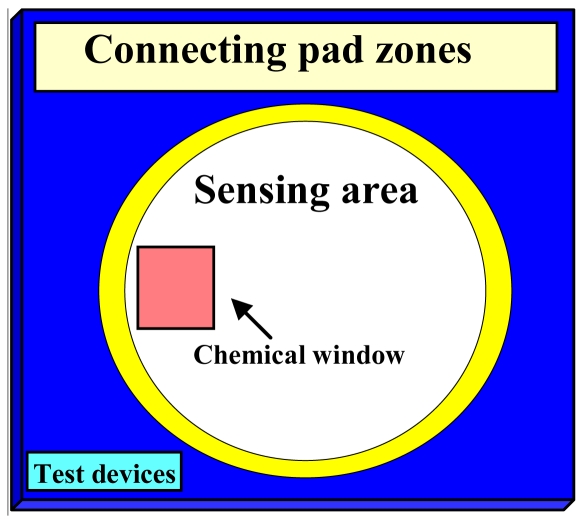
Schematic illustration of die layout (17 mm × 17 mm).

In the overall die layout (Figure 1), the central gold circle defines the sensing area, and the location of the sealing O-ring of the liquid flow-cell. The lower part contains the test structures zone. On the left side, a chemical window (1.5 mm × 1.5 mm) is located that was used in order to perform detailed surface analysis (AFM and ellipsometry).

### 2.2. Electronic Measurements

**Figure 2.**
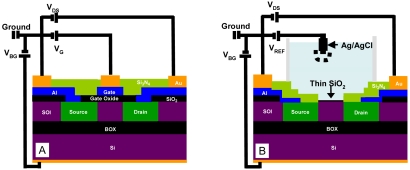
Schematic cross-sections of A) test structure and B) EISFET. The gate oxide thickness of the test structure is ∼ 100 nm, while the gate oxide thickness of the EISFET is ∼ 30. Both devices are based on thin, 10 to 40 nm SOI layer. The source/drain regions are connected through metallization which in turn is protected by passivation layer. The distance between the drain and the source defines the channel length.

**Figure 3.**
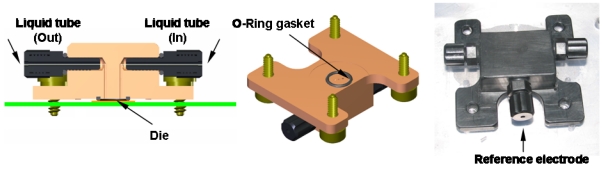
Liquid flow-cell design and manufacture. Left: cross-section illustrating the inlet and the outlet tubing entrances. The silicon die is located at the bottom of the flow-cell held tight using 4 thumbscrews (middle picture). The O-ring holds the die in close proximity with the flow-cell, maintaining all liquids within the die ‘wet’ area far away from the contact pads. The right picture is the actual flow-cell with its reference electrode entrance. This flow-cell design includes the following unique features: low sample volume (30 μL), ease of sample replacement, light protecting and chemically stable material.

I-V measurements were performed for both test structures and FD EISFETs. For the FD EISFETs, I-V measurements were performed both under dry and wet conditions. The electrical setting for both the test structures and the FD EISFETs wet measurements are presented in Figures 2A, B, respectively. In order to work in aqueous conditions we designed a unique liquid application apparatus - a flow cell (Figure 3). This unique design facilitated the work in aqueous environments without the need for contact isolation. The liquids were retained within the O-ring gasket while the thumb screws were tightened against the probe-station chuck. The connecting pads were left out, providing easy approach for electrical testing (see also Figure 1). The apparatus included additional important features; ultra-low sample volume (30 μL), fast and convenient way of die replacement and black material to prevent light induced currents. A homemade Ag/AgCl wire type reference electrode (*V*_REF_) was used for the wet I-V measurements that were performed with various solutions. I-V measurements of the test structures and the dry FD EISFETs were performed with an analog tester (Agilent – HP4062) and automatically probed by an Electroglass EG2010 probe.

### 2.3. Device Functionalization

After a brief dip in distilled water (18 Mohm) and drying under streaming N_2_, devices were activated by G-1000 Oxygen down stream plasma (Yield Engineering Systems Inc., San Jose, CA, USA), and immediately modified for the specified time in ethanol:water (95:5) solution containing 1%(v/v) aminopropyltrimethoxysilane (APTMS, Gelest Inc., Morrisville, PA, USA). The devices were then washed with ethanol, dried over N_2_, and kept desiccated in closed vial for further measurements (not longer than 24 hours). Surface response to various treatments (Figure 10B) was monitored as previously described [23]. Briefly, devices were activated using ultraviolet ozone cleaning system (UVOCS, T10X10, Montgomeryville, PA) followed by APTMS monolayer formation. The sample was then immersed in strong acid, dried, and measured, followed by the same process but with strong base.

### 2.4. Contact Angle Analysis

A contact angle meter (Ramé-Hart instrument co. Netcong, NJ, USA) was used to measure the static water contact angle of the films. Contact angles of 4 μL water drops were measured according to a standard method [24] during surface modification at room temperature. The reported results are the average of six different samples taken at three different spots of a given sample.

### 2.5. pH Measurements

Following 4 min of APTMS activation (described in a previous section) the devices were inserted into the custom-made flow-cell for pH measurements. The flow-cell was then washed with 2 mL of 10 mM phosphate buffer (PB) with pH value of 6.2 in order to initiate the experiment. The device was subjected to 5 mL of PB solution with different pH values (pH: 6.2, 6.7 and 8.2). The source-drain current (*I*_DS_) was measured continuously and the modulation of *I*_DS_ with the various pH levels was recorded. In order to translate the *I*_DS_ modulation into pH sensitivity in terms of mV/dec, a 10 mV calibration pulses were given at the relevant pH levels in order to extract the FD EISFET gain (*g_m_*). The time-based pH measurements were performed in the linear region with *V*_DS_ = 1 V, *V*_REF_ = 1.5 V and *V*_Gb_ = 50 V.

### 2.6. AFM Surface Analysis

The topographies of the thin films were investigated by AFM (Solver P47 PRO Scanning Probe Microscope). AFM was operated in contact mode in air and at room temperature. Images were acquired at a scan rate of 1.5 Hz with a silicon cantilever (Ultralever 06 B, PSI, USA), and the scan size used was 1 μm × 1 μm.

## 3. Results and Discussion

### 3.1. Fabrication

**Figure 4.**
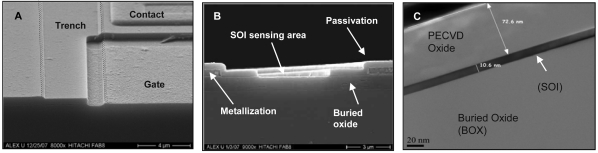
SEM and TEM images of device cross-sections. (A) A tilted top view of the sliced device. (B) Side view cross-section of the same device. Sensing area is shown in the middle surrounded by drain/source regions connected to metallization layer. (C) Side view TEM cross section showing relative thickness of the different layers that are illustrated in Figure 2.

**Figure 5.**
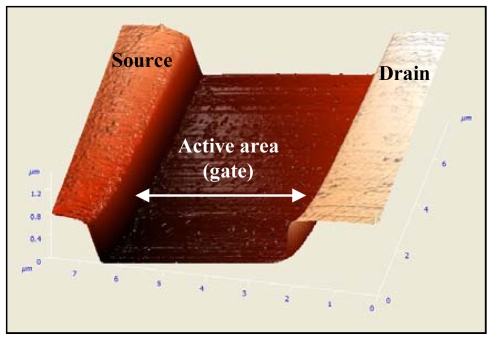
AFM tapping mode topographic image of the active gate area.

Figures 2A and 2B depict schematic cross-sections of the test structure and FD EISFET, respectively, with their basic layers. The source/drain regions are connected through metallization which in turn is protected by passivation layer as can be seen in Figure 4. The role of the passivation layer is to avoid shorts and to protect the metallization from chemical solution attack. Due to the shallow SOI layer the drain and source regions extend vertically down to the buried oxide. The clean-room process steps of the chemical window are identical to the FD EISFET active area (gate). Figures 4A and B illustrates representative SEM cross-section where the sensing area is located in the middle surrounded by drain/source regions connected to metallization. The metallization layer is covered by the passivation nitride layer. TEM analysis (Figure 4C) demonstrates the various layers in the device including a device with a thin ∼ 10 nm SOI layer. The last process step (opening the active gate area) was monitored using AFM roughness analysis (Figure 5) indicating that this step did not resulted with a significant roughness increase maintaining RMS roughness of 0.62 nm.

**Figure 6.**
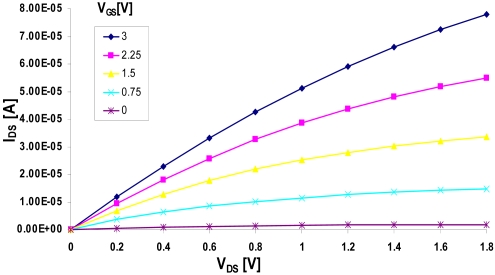
I-V characterization of a typical test structure. *I*_DS_ versus *V*_GS_ of a depletion type (n-type) device fabricated with a 10 nm SOI layer.

Test structures were fabricated in order to monitor and evaluate the performance of the clean room fabrication, and also provide means by which standard and automatic CMOS electrical testing (ETEST) can be performed. Figure 6 presents the I-V curve of an n-type rectangular-shaped test structure with a channel width and length of 20 μm and 5 μm, respectively. The back-gate (*V*_Gb_) voltage was set to 0 V, the drain-source voltage (*V*_DS_) was swapped between 0 and 1.8 V with 0.2 V intervals, and the front gate voltage was swapped between 0 V and 3 V with 0.75 V intervals. It is demonstrated that the threshold voltage (*V*_T_) and transconductance (*g*_m_) are ∼ 0 V and ∼ 29 μA/V respectively. Accordingly, the device is a depletion type device with a relatively high *g*_m_. The test structure performance indicated that the thin SOI layer retains its single crystal properties throughout processing.

### 3.2. Electrical Testing

**Figure 7.**
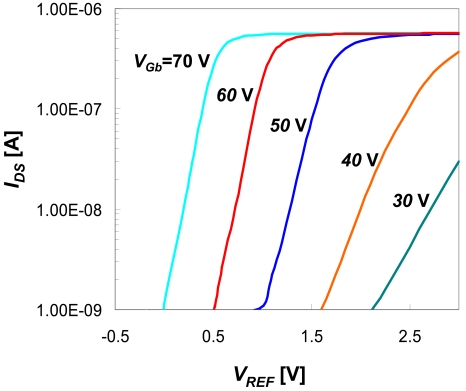
*I*_DS_ vs. *V*_REF_ of an EISFET for various values of *V*_Gb_. Note the shift in front threshold voltage with increasing *V*_Gb_ (*V*_DS_ = 0.5 V).

I-V electrical characterization of the FD EISFET was performed pre surface modification. Representative *I*_DS_ vs. Ag/AgCl reference electrode voltage (*V*_REF_) at pH 7 for different back gate voltage (*V*_Gb_) are presented in Figure 7. The width (W) and length (L) of the measured FD EISFET are 100 μ and 10 μ, respectively. Note that higher values of *V*_Gb_ imply reduction in front threshold voltage (*V*_Tf_). This *V*_Tf_ dependency upon *V*_Gb_ is a manifestation of the existing charge coupling between the front and back interfaces in FD SOI. The continuous decrease in *V*_Tf_ for increasing *V*_Gb_ reflects the transition of the back interface from accumulation into inversion. More specifically, the more depleted the back interface, the lower is *V*_Tf_. For inverted back interface, *V*_Tf_ is lowest and constant, and for accumulated back interface *V*_Tf_ is highest and constant [22,25]. Note that inversion and accumulation of the back interface are not apparent in the present case. A more comprehensive descriptions of the basic properties of the sensor device can be found in our previous publications [22,23,26].

### 3.3. Activation

Choosing optimal activation conditions are of significant importance for FET-based biosensors. Parameters like surface morphology and self-assembly film activity depends on many factors such as reagent concentration, type of solvent and water percentage. The most common and successful route for the deposition of self assembled monolayer (SAM) on Si substrates is through silanization process. In this process, SAMs are formed spontaneously by immersing the OH-terminated SiOx/Si substrates into an active solution. The mechanism of SAM formation process is well established, which is known to take place in four steps: 1) physical adsorption onto the hydrated silicon surface, 2) hydrolysis of the silane head-groups (SiX_3_) in the presence of the adsorbed water layer on the surface into highly polar silanetriol Si(OH)_3_, 3) covalent bonds formation of the silanetriol with the silanol groups (Si-OH) on substrate surface, and 4) self-organization of the monolayer driven by van der Waals interactions among the linear alkyl-chains into a packed dense layer [27-29]. During the initial stage (short incubation times), only a few molecules will adsorbed on the substrate surface and it is expected that the monolayer will be in a disordered (or liquid) state. However, at longer incubation times, the surface coverage eventually reaches the point where a well-ordered and compact monolayer is obtained. It was also demonstrated that the growth kinetics depends on the alkyl-chain length that is a result of chain length dependent chemisorption and diffusion rates of molecules on the substrate surface, which are known to decrease with increasing chain length [30].

**Figure 8.**
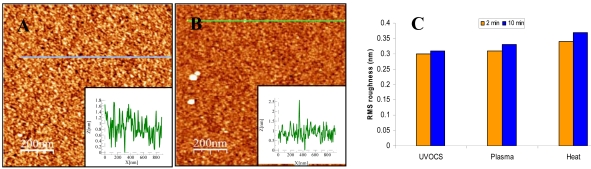
AFM tapping mode topographic images of modified surfaces. Surface analysis was done following 20 min UVOCS and 2 min (A), or 10 min (B), APTMS (aminopropyltrimethoxysilane) monolayer formation. Topographical scans were done in non contact mode with Si_3_N_4_ pyramidal tips with tip curvature of ∼ 15 nm, force constant (k) ∼ 0.5 N/m, and with scan rate of 0.5Hz. The data obtained for A was: RMS roughness: 0.3 nm, average height: 1.07 nm, Min/Max: 0.77 nm, and for B: RMS roughness: 0.31 nm, average height: 0.94 nm, Min/Max: 2.52 nm. (C) RMS roughness comparison analysis for different pre-activation conditions.

**Figure 9.**
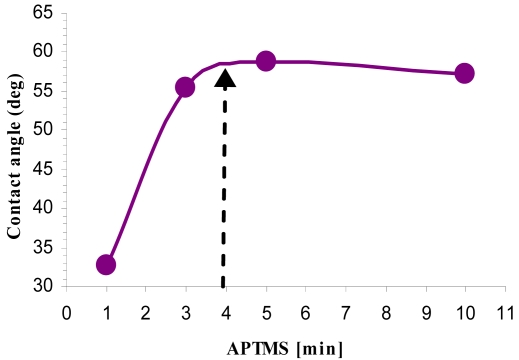
Correlation between contact angle and APTMS modification time (1, 3, 5 and 10 min). Working conditions chosen for device modification are indicated by dashed arrow. This figure illustrates the graduate changes (increase) in contact angle as the monolayer (APTMS) deposition time increases.

Our data presented in Figures 8 and 9 supports this model, demonstrating that by changing pre-activation conditions, modification time and reagent mixtures, we could control saturation curves in single degree resolution. Typical AFM images of the samples prepared using fixed APTMS concentration in various incubation times had demonstrated a height profile with a uniform coverage of the substrate with a surface roughness < 1 Å (Figures 8A and B). We also demonstrated that the pre-activation conditions have small effect on the RMS roughness (Figure 8C). This analysis verify our procedure to be one that results with very smooth film with low RMS roughness which is similar to the roughness of the silicon surface itself. Contact angle provided a complementary method for SAM characterization since it is very sensitive to the differences between the hydrophobic natures of the alkyl chain versus the hydrophilic nature of the animated head group.

The measured DI water contact angles indicated the direct correlation between incubation time and contact angle up to a level of a uniform layer (Figure 9). It is important to note that the reported water contact angles for NH_2_-terminated surfaces are highly variable and data between 15 and 68 are reported [31,32]. This discrepancy is probably a result of the high activity of NH_2_ head group adsorbing impurities from the atmosphere in the time duration between SAM formation and contact angle measurements [33,34]. We have concluded that shorter incubation time resulted with non-uniform monolayers whereas longer time duration resulted with better surface coverage and a more uniform layer. The chosen working conditions represent “mid-point” of full coverage APTMS self assembly similar to what is reported in literature (Φa ∼ 42° to 68°) (Figure 9) [35].

### 3.4. pH Sensing

**Figure 10.**
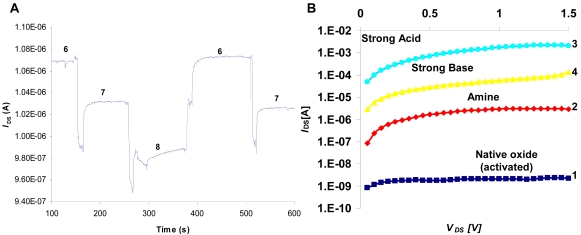
(A) pH sensitivity of APTMS activated depletion type (n-type) transistor with a 10 nm thin Si layer. *I*_DS_ vs. time was taken for different pH values (the working point was set to the linear region at: *V*_DS_ = 1V, *V*_REF_ = 1.5V, *V*_Gb_ = 50V). In order to determine pH sensitivity, the *I*_DS_ current was divided by *g*_m_ (calculated using calibration pulse). (B) Demonstrating device performance following APTMS activation. *V*_Gb_ was held constant at 50 V. The base line is the I-V curve for device covered with activated native oxide (1). Following APTMS monolayer formation, a significant increase in currents can be detected (2). Incubating the device in strong acid (APTMS head group protonation) resulted with additional current boost (3). Consequently, head groups de-protonation using strong base, resulted in current decrease (4).

We have used our flow-cell apparatus in order to monitor on-line device response to pH alteration presenting the results over time (Figure 10A). The drain current increased or decreased depending on the pH value. The APTMS activated, n-channel device demonstrates decreasing *I*_DS_ for increasing pH values, as expected. A pH sensitivity of 40 mV/pH is recorded which is lower than the theoretical value for silicon oxide in this pH range (47 mV/dec for pH 6 and 51 mV/dec for pH 8). Since the presented data is of APTMS activated surfaces, we anticipated that the theoretical value will not be obtained. Furthermore, considering the mixed amphoteric surface, our results indicate on very sensitive devices. In addition, these devices demonstrated stable pH sensitivity in electrolyte solution or in dry condition over time up to several months (data is not presented).

The pH sensitivity of amphoteric surfaces depends upon the intrinsic buffer capacity of the surface and the capacitance of the double layer created at the dielectric/solution interface. The intrinsic buffer capacity is described by the site-dissociation model that attributes the processes of protonation and deprotonation of titratable surface groups (e.g. amphoteric OH groups on SiO_2_ gate dielectric). The capacitance of the double layer is a function of the salt concentration and the surface potential [36]. Protonation/deprotonation processes are the key factors dominating device pH sensitivity through its significant impact on the electrochemical characteristics of amphoteric groups. In the present case, the mixed interface is composed of two amphoteric groups: the −SiOH with pKa value of 6.8 and −NH_2_ with pKa value of 9 – 10 (attributed to APTMS amino group). Therefore it is anticipated that the interface is fully protonated at pH in the range of 6.8 to 9 [37-39]. The observed linear response (Figure 10A) can be attributed to an approximately linear change in the total surface charge density (versus pH) because of the combined acid and base behavior of both surface groups [40]. In order to further illustrate this phenomenon we plotted device I/V curves following alteration of acidic and basic treatments (Figure 10B). As a base line we measured device performance following surface activation. The next step was APTMS monolayer modification that resulted with significant increase in drain current that can be attributed to positive charge accumulation. Protonation of the amine head group under acidic condition (transition from −NH_2_ to −NH_3_^+^) resulted with further accumulation of positive charge on the surface. Deprotonation of the amine group under basic condition resulted with decreased drain currents.

### 3.5. Sensing Biomolecular Interactions

**Figure 11.**
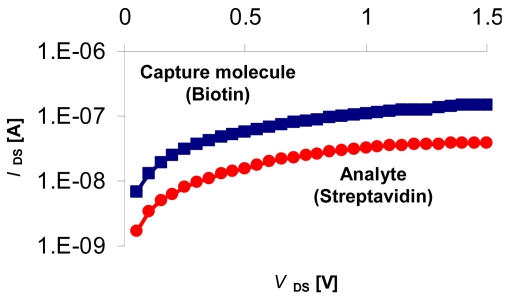
First label-free results from bio-sensing device based on the FD EISFET platform. The first structure analyzed for analyte detection was tested on a biotin-streptavidin model system. Results indicate that we can detect analyte (20 nm streptavidin) interacting with the capture molecule (biotin).

To investigate biomolecular interaction monitoring, we functionalized the gate surface with biotin and studied the well-known binding of biotin to streptavidin (Figure 11). The measurement was taken at the subthreshold regime with *V*_Gb_ = 50 V, *V*_DS_ = 50 mV and *V*_REF_ = 1 V, in 50 mM phosphate buffer pH 7. Our measurements demonstrate a clear signal following the addition of 20 nm streptavidin. The observed decreased currents are consistent with binding of a negatively charged species to the activated gate surface, and the fact that streptavidin is a negatively charged molecule at the current working conditions (pI: 5.5). In addition, the stable signal is the result of the nearly covalent biotin-streptavidin binding (Ka ∼ 10^-15^ M^-1^) [41]. The proposed mechanism for the sensing activity is that linking negatively charged molecule to the active gate area is equivalent to negative gate biasing resulting with changed surface potential that eventually modulate channel currents. The charge coupling between the front and back interfaces in FD EISFET and the subsequent ability to optimize the sensitivity of FD EISFET to surface potential variations was already demonstrated [22,23]. The current label-free measurement was executed at the subthreshold regime where the FD EISFET sensitivity is greater for higher *V*_GB_. Finally, drift measurements of this system was in the range of ∼ 3 mV/h^-1^, which is consistent with drift values cited in current literature [42]. As the length of measurement did not exceed 15 min, the effect of drift current is negligible in comparison with the current increase due to strepavidin detection.

## 4. Conclusions

Here we present the first step into the fabrication of a low cost sensitive device with potential future application for bio-sensing. Successful exploitation of this exciting possibility is critically dependent on a high volume manufacturing potential that will result in a low unit cost. We expect that the high *g*_m_ values and the high pH sensitivity will lay the foundations for the detections of diverse biomolecular interactions.
